# Characterization of TRPV2-associated pathology in Parkinson’s disease and therapeutic modulation using Tranilast

**DOI:** 10.3389/fncel.2026.1896592

**Published:** 2026-08-06

**Authors:** Apeksha Chunchuwar, Bhupesh Vaidya, Shyam Sunder Sharma

**Affiliations:** Department of Pharmacology and Toxicology, National Institute of Pharmaceutical Education and Research, Mohali, India

**Keywords:** 6-OHDA, Parkinson’s disease, Tranilast, TRPV2, tyrosine hydroxylase

## Abstract

**Introduction:**

Transient Receptor Potential (TRP) channels have gained worldwide attention for their expression and distribution in multiple human organs. Among these TRPs, Transient Receptor Potential Vanilloid (TRPV) channels play diverse physiological roles in the brain, and changes in their expression contribute to central nervous system (CNS) diseases. In a previous druggable-genome α-synuclein modifier screen, a member of the TRPV family, TRPV2, emerged as a Tier 3 ion-channel candidate, meeting directionality and conflict-score criteria, though with limited shRNA-level support. However, based on this preliminary data and the known role of TRPV2 in Ca^2+^ entry and cellular stress responses, we decided to investigate its role in detail using experimental PD models.

**Methods:**

In the present investigation, 6-hydroxydopamine (6-OHDA) was used *in vivo* (Sprague Dawley rats) and *in vitro* (SH-SY5Y cells) to induce Parkinson’s disease (PD), and its effect on TRPV2 expression was investigated. The study further involved measuring behavioral parameters using the rotarod, open field, apomorphine, and cylinder tests.

**Results:**

6-OHDA administration induced excessive reactive oxygen species (ROS) production and increased expression of TRPV2 channels. This is also accompanied by increased intracellular calcium influx and reduces cell viability. However, Tranilast, a TRPV2 antagonist, exhibited promising neuroprotective effects in the 6-OHDA PD model by restoring tyrosine hydroxylase levels, reducing oxidative stress, and improving motor function. Also, it diminished the intracellular Ca^2+^ influx and reduced ROS levels in the 6-OHDA-treated SH-SY5Y cells.

**Conclusion:**

Overall, our results suggest that TRPV2 levels are elevated in PD, and targeting TRPV2 could be a potential therapeutic approach in the future.

## 1 Introduction

Parkinson’s disease (PD) is the second most prevalent neurodegenerative disease in the world. The death of tyrosine hydroxylase-positive dopaminergic neurons is a major event in PD pathology and contributes to its motor and non-motor symptoms. However, this decreased level of dopamine is the result of an alteration of multiple cellular events that precede neuronal death, such as neuroinflammation, mitochondrial dysfunction, elevated oxidative and nitrosative stress, and impaired autophagic or proteasomal protein degradation (Barata-Antunes et al., 2020; Prasad and Hung, 2020; Desai et al., 2019). Despite the complex pathophysiology and multiple pathways affected in PD, the main treatment options are still aimed at increasing dopamine levels to improve motor deficits. Unfortunately, this approach does not present a long-term solution and hence medications tend to lose their efficacy as dopaminergic neurodegeneration progresses over time. Therefore, PD is still incurable and needs more precisely targeted therapies to stop or slow down the disease progression (Ellis and Fell, 2017; Mao et al., 2020).

In this regard, targeting transient receptor potential (TRP) channels could open new avenues for the development of PD therapeutics. TRP channels are non-selective cation channels that regulate physiological functions in response to diverse environmental changes and stimuli (Wang et al., 2020). TRP channels are expressed throughout the body, including the brain, heart, kidneys, and lungs. Even within the brain, several TRP channels are expressed in both neurons and non-neuronal cells, including glia, and have a role in neurogenesis. Therefore, any changes in TRP expression could impair these processes and contribute to neurodegenerative diseases such as PD and AD (Duitama et al., 2020). Among the TRP channel family, Transient receptor potential vanilloid 2 (TRPV2) is a calcium-permeable cation channel. This channel is activated in response to temperatures >52 °C, exogenous and endogenous ligands, and mechanical stressors. A large portion of TRPV2 is also located in the endoplasmic reticulum. However, stimulation of the cells with phosphatidylinositol 3-kinase-activating ligands is responsible for its translocation to the plasma membrane, where it functions as a cation channel (Kojima and Nagasawa, 2014; Luo et al., 2021) and it may exacerbate neuronal injury under pathological conditions.

Notably, a previous PD druggable-genome α-synuclein modifier screen identified TRPV2 as a Tier 3 ion-channel candidate that met directionality and conflict-score criteria, suggesting a potential association with PD-relevant cellular pathways (Rousseaux et al., 2018). Collectively, these observations position TRPV2 as a potentially druggable convergence point linking calcium dyshomeostasis, oxidative stress and mitochondrial dysfunction in PD.

This evidence is further supported by the fact that Ca^2+^ dyshomeostasis results in excitotoxicity, which has been linked to the progression of neurodegenerative disorders, including AD and PD (Thapak et al., 2020). Excessive Ca^2+^ loads can eventually lead to cell death by aberrant activation of processes that normally operate physiologically at low levels, causing metabolic impairments (Naziroglu, 2012). Therefore, targeting key physiological pathways responsible for calcium imbalance could help maintain cellular homeostasis and reverse pathological symptoms observed in neurodegenerative disorders, including PD. In line with this, the present study investigated Ca^2+^-dependent pathogenic factors leading to PD by blocking this channel using Tranilast in the 6-OHDA model.

## 2 Materials and methods

### 2.1 Animal experiments

Male Sprague Dawley rats (250–300 g) used in this study were housed in a room at 21 ± 1 °C and 55% ± 5% humidity, under a 12-h light/dark cycle, with food and water available *ad libitum*. All animal experiments were conducted in compliance with the Institutional Animal Ethics Committee of the National Institute of Pharmaceutical Education and Research, S.A.S. Nagar, India, as well as the ARRIVE guidelines.

### 2.2 Induction of PD

For unilateral intrastriatal injection of 6-OHDA, the rats were first anesthetized with thiopental sodium (50 mg/kg). Rats were injected with 2 μl of 6-OHDA (4.86 mg/ml 6-OHDA in 0.02% ascorbic acid and 0.9% NaCl), and sham rats were injected with a physiological solution (0.02% ascorbic acid in 0.9% NaCl) at each injection site. The two unilateral injections were localized in the right dorsal striatum at coordinates (A+0.5; L+2.5; P-5) and (A-0.5; L+4; P-5) (Perlbarg et al., 2018). Animals were then randomized and divided into different groups comprising Sham animals (surgery controls), 6-OHDA-administered animals, 6-OHDA-administered animals treated with either Tranilast (30 or 100 mg/kg) or vehicle and the naïve control animals treated with Tranilast (100 mg/kg) (*Per se*). Sample sizes for each experiment were determined using power analysis based on our previous investigations involving TRP channels (Vaidya et al., 2022a,b; Thapak et al., 2022).

### 2.3 Treatment schedule

**FIGURE 1:**
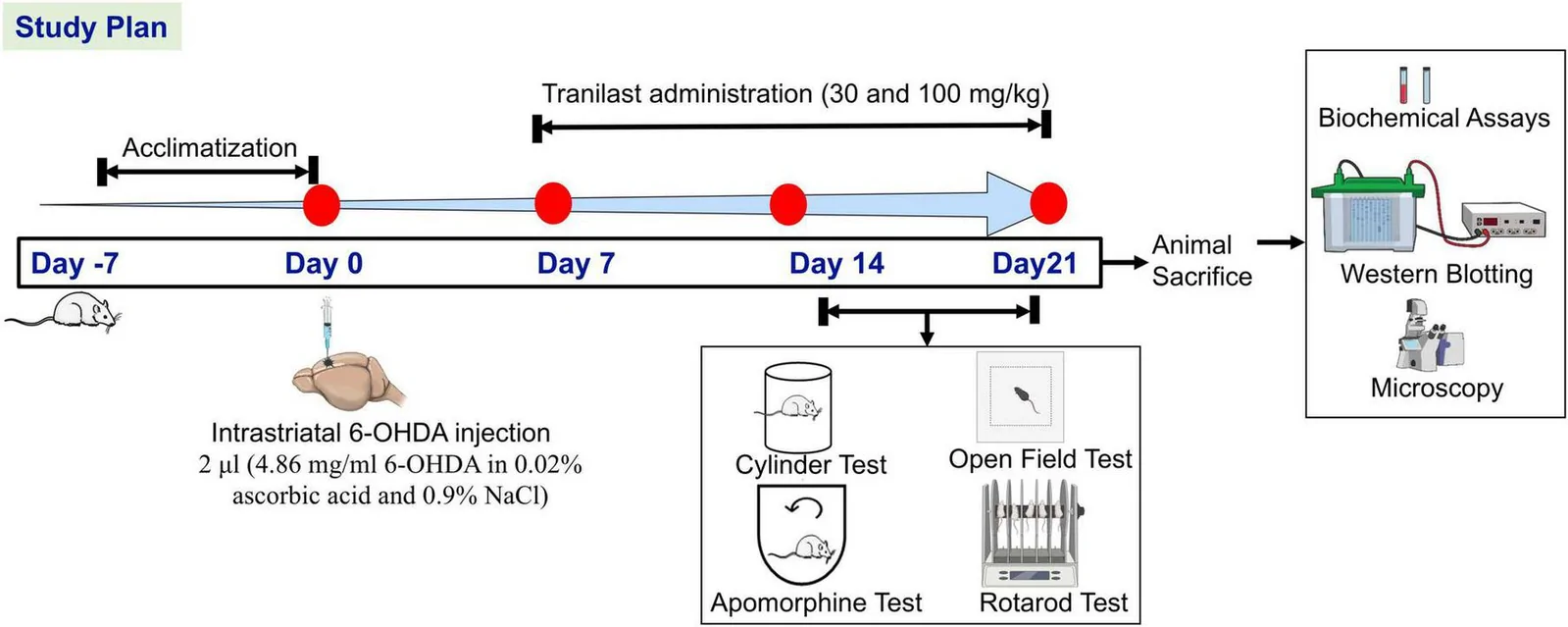
Schematic representation of *in vivo* experimental design and treatment schedule. Unilateral 6-OHDA was administered intrastriatally on day 0. Assessment of all the behavioral parameters was made between days 14 and 21. Open field activity (day 14), cylinder test (day 15), rotarod test (days 16–19) and apomorphine test (day 20). This was followed by the sacrifice of animals by decapitation on day 21. All the biochemical parameters, histological examinations and other assessments were made after the sacrifice of animals.

Tranilast (30 and 100 mg/kg) was administered by oral gavage 1 week after unilateral intrastriatal administration of 6-OHDA. Doses of Tranilast were selected based on previous investigations (Thapak et al., 2020, 2022). Drug administration was continued for 2 weeks while behavioral parameters were evaluated during the third week as shown in Figure 1.

### 2.4 Rotarod test

A rotating rod apparatus (ROTA ROD, Ugo Basile) was used to assess the motor performance of the rats. Rats were trained with four trials per day for three consecutive days at 15 rpm. Each trial lasted up to 60 s. On the fourth day, a single trial was performed in which rats were tested at accelerating speeds from 4 to 40 rpm over a 300-s duration. Fall latency and rpm of fall were recorded and compared across groups (Sarukhani et al., 2018; Campos et al., 2013).

### 2.5 Open field test

The open field test was performed as described previously (Vaidya et al., 2022a). Rats were allowed to explore the open field for 10 min. Open-field activity was measured using ANY-maze software and compared across groups.

### 2.6 Apomorphine-induced rotations

Apomorphine-induced rotational behavior results from a nigrostriatal dopaminergic imbalance between the right (lesioned) and left (unlesioned) brain hemispheres. In this test, animals were placed inside a cylindrical container having a diameter and height of 28 and 38 cm, respectively, and were habituated for 5 min followed by an injection of apomorphine hydrochloride (0.5 mg/kg, i.p. in saline). The contralateral rotations (opposite to the lesioned side) and the ipsilateral rotations (toward the lesioned side) were monitored and counted for an hour (Ximenes et al., 2015; Sarukhani et al., 2018).

### 2.7 Cylinder test

Rats were individually placed in a transparent cylinder (19 cm diameter, 25 cm height) and recorded for 5 min to measure spontaneous forelimb use while exploring the cylinder wall. The video was later analyzed in slow motion to score the movements over 5 min. The number of supporting wall contacts of the right and left forelimb during lateral movements as well as rearing along the wall was quantified (Barata-Antunes et al., 2020; Shaqfah et al., 2025).

### 2.8 Cresyl violet staining

Cresyl violet was used to determine neuronal cell viability following staining of Nissl granules. Briefly, the paraffin-embedded brain sections were deparaffinized with xylene for 5 min, then rehydrated for 3 min in a gradient of alcohol solutions (95% and 70%). Afterward, slides were washed with deionized water and incubated for 15 min in 0.5% cresyl violet at 60°C. Subsequently, the slides were rinsed with increasing alcohol gradients (70%, 90%, and 100%) and finally mounted using DePeX. Images were taken using the brightfield microscope (Leica DFC320) (Sushma et al., 2023; Asle-Rousta and Peirovy, 2024).

### 2.9 Immunofluorescence

Immunofluorescence was performed as described earlier (Singla et al., 2026). Briefly, 5 μm paraffin-embedded sections were cut with a microtome, cleared in xylene, and washed with a graded alcohol series. Subsequently, antigen retrieval was performed using citrate buffer (pH 6), and sections were blocked with 2.5% horse serum. The sections were then incubated overnight with the anti-TRPV2 antibody (Abcam, Cat# 106829; 1:50). The sections were washed the following day and incubated again with the fluorophore-tagged secondary antibody. The sections were washed again and incubated with DAPI. After the final wash, the sections were mounted using glycerol and imaged under a microscope. Images were later quantified using ImageJ.

### 2.10 Western blotting

Protein extracts of the striatum were prepared by homogenizing in lysis buffer [Tris- HCL 50 mM, SDS 10%, Sodium deoxycholate 10%, PMSF 1 mM, EDTA 5 mM, EGTA 1 mM, Sodium fluoride (NaF) 5 mM, Sodium ortho vanadate 1 mM, Cocktail (protease inhibitor) 1 ul/ml, Sodium chloride (NaCl) 150 Mm, Triton X-100 1%]. The supernatant was collected by centrifuging the homogenate at 12000 rpm for 10 min. Proteins were separated using SDS-PAGE and transferred onto a nitrocellulose membrane. Membranes were blocked with 5% (w/v) skimmed milk in TBS-T then incubated overnight at 4 °C with antibodies against TRPV2 (Abcam, Cat# 106829; 1:500), β-actin (Sigma-Aldrich, Cat#; 1:1000) and TH (Sigma-Aldrich, Cat# T2928; 1:6000). Subsequently, the following day the expressions were detected with horseradish peroxidase (HRP) conjugated secondary antibodies (1:10000) by the enhanced chemiluminescence method. The band intensities were quantified with the help of ImageJ software and normalized by β-actin (Panghal et al., 2022; Rihan and Sharma, 2024).

### 2.11 Immunohistochemistry

To analyze the effects of the 6-OHDA lesion and Tranilast at the histological level, immunostaining for tyrosine hydroxylase (TH) was performed in the right striatum. For this, the entire brain was fixed by transcardial perfusion performed under anesthesia (thiopental sodium 50 mg/kg i.p.) with 4% paraformaldehyde in PBS. Then the brains were processed for paraffin embedding and 5 μm were taken. The sections were deparaffinized and hydrated with graded concentrations of alcohol followed by incubation in boiling citrate buffer for antigen retrieval. Sections were then incubated with the peroxidase blocking agent and horse serum solution before the sections were incubated overnight at 4 °C with the primary antibodies for TH (Sigma Aldrich, Cat# T2928; 1:75 dilution). The sections were washed again with TBS and incubated with the secondary antibody followed by polymer solution for 30 min. At last, sections were treated with chromogen DAB (3,3′-Diaminobenzidine) and mounted with 90% glycerol to observe the sections under the brightfield microscope (Nikon SMZ1500). Finally, the slides were analyzed under the brightfield microscope (Leica DFC320) at 20× magnification by mounting the sections with the help of 90% glycerol (Vaidya et al., 2022a).

### 2.12 Malondialdehyde (MDA) estimation

Malondialdehyde estimation was performed after tissue homogenization in PBS at a 1:10 ratio. Subsequently, SDS (8.1%, 100 μl), acetic acid (20%, 750 μl), and TBA (0.8%, 750 μl) were added to 100 μl of tissue homogenate. It was boiled for 1 h at 95°C, centrifuged briefly for 10 min, and the absorbance was measured at 532 nm. Protein estimation was done using the Lowry method and MDA levels were normalized to the mg of protein in the samples (Singla and Jena, 2022; Gala et al., 2025).

### 2.13 Glutathione (GSH) estimation

Glutathione estimation was done using Ellman’s reagent. The sample supernatants were mixed with phosphate buffer (pH 7.4), and Ellman’s reagent was added to it. The mixture was incubated at 38°C for 10 min, and the absorbance was measured at 415 nm. Protein estimation was done using the Lowry method and GSH levels were normalized to the mg of protein in the samples (Panghal et al., 2022; Ellman and Lysko, 1979).

### 2.14 Cell culture

Human SH-SY5Y cells (purchased from the National Centre for Cell Science Pune, India) were grown in Dulbecco’s modified Eagle’s medium (DMEM) medium supplemented with L-glutamine, 10% FBS, and 1% antibiotic solution at 37°C in 95% humid air and 5% CO_2_. Cells were sub-cultured at 80% confluency by adding trypsin- EDTA 0.05% in a T-25 flask (Jahanshahi et al., 2018; Vaidya et al., 2022b).

### 2.15 MTT assay

3-(4, 5-Dimethythiazol-2-yl)-2,5-diphenyl tetrazolium bromide (MTT) was used to assess cell viability. Initially, to check the dose-dependent effect of 6-OHDA, SH-SY5Y cells were exposed to different concentrations of neurotoxin 6-OHDA (25, 50, 75, 100,150 μM) for 24 h. A dose-response curve was plotted for the same and a 75 μM concentration was selected for further experiments. Also, the cells were then co-treated with different concentrations of Tranilast (0.01, 0.03,0.1, 0.3, and 1 mM) for 24 h to determine the effective dose. The next day, the media was removed, and MTT (0.5 mg/ml; 100 μl) was added to the wells. Afterward, the MTT solution was removed and DMSO (100 μl) was added to solubilize the crystals of formazan. Finally, the absorbance was noted at 570 nm wavelength using a multi-well plate reader (μ-Quant, Biotek instruments) and compared across groups (Massari et al., 2016; Jahanshahi et al., 2018).

### 2.16 H_2_DCFDA assay

The H_2_DCFDA assay was performed to measure the reactive oxygen species (ROS). Cells were seeded onto coverslips and placed in a 6-well plate. The following day, each well was exposed to different treatment groups for 24 h. The next day cells were washed with DPBS and incubated with H_2_DCFDA (10 μM) for 45 min. After 45 min cells were washed with DPBS and fixed. Finally, coverslips were washed with DBPS and mounted on glass slides using 90% glycerol. Images were taken at 485/528 nm (excitation/emission) wavelength using the fluorescent microscope (Leica DFC320) (Vaidya et al., 2023; Ahmed et al., 2025).

### 2.17 Fura 2-AM assay

Intracellular [Ca^2+^] influx was measured using fluorescent Fura 2-AM dye. Cells were seeded in a 96-well plate and incubated at 37°C in 95% humid air with 5% CO_2_. After 24 h cells were subjected to different treatments and FURA-2-AM (5 μM) was added to each well. The baseline absorbance of the wells was measured using a spectrofluorometer (SPECTRAmax) at the excitation wavelength (340/380 nm) and emission wavelength (510 nm). Change in fluorescence was again measured after 24 h and compared across groups (Massari et al., 2016; Heusinkveld and Westerink, 2017).

### 2.18 Statistical analysis

Data were expressed as Mean ± SEM. Data was checked for normality and statistical comparison between the groups was performed using one way analysis of variance followed by Tukey’s post-hoc analysis. Statistical outliers, if any, were excluded using the Grubbs’ test. *p* < 0.05 was considered as statistically significant.

## 3 Results

### 3.1 Effect of Tranilast (30 and 100 mg/kg) on behavioral parameters

**FIGURE 2:**
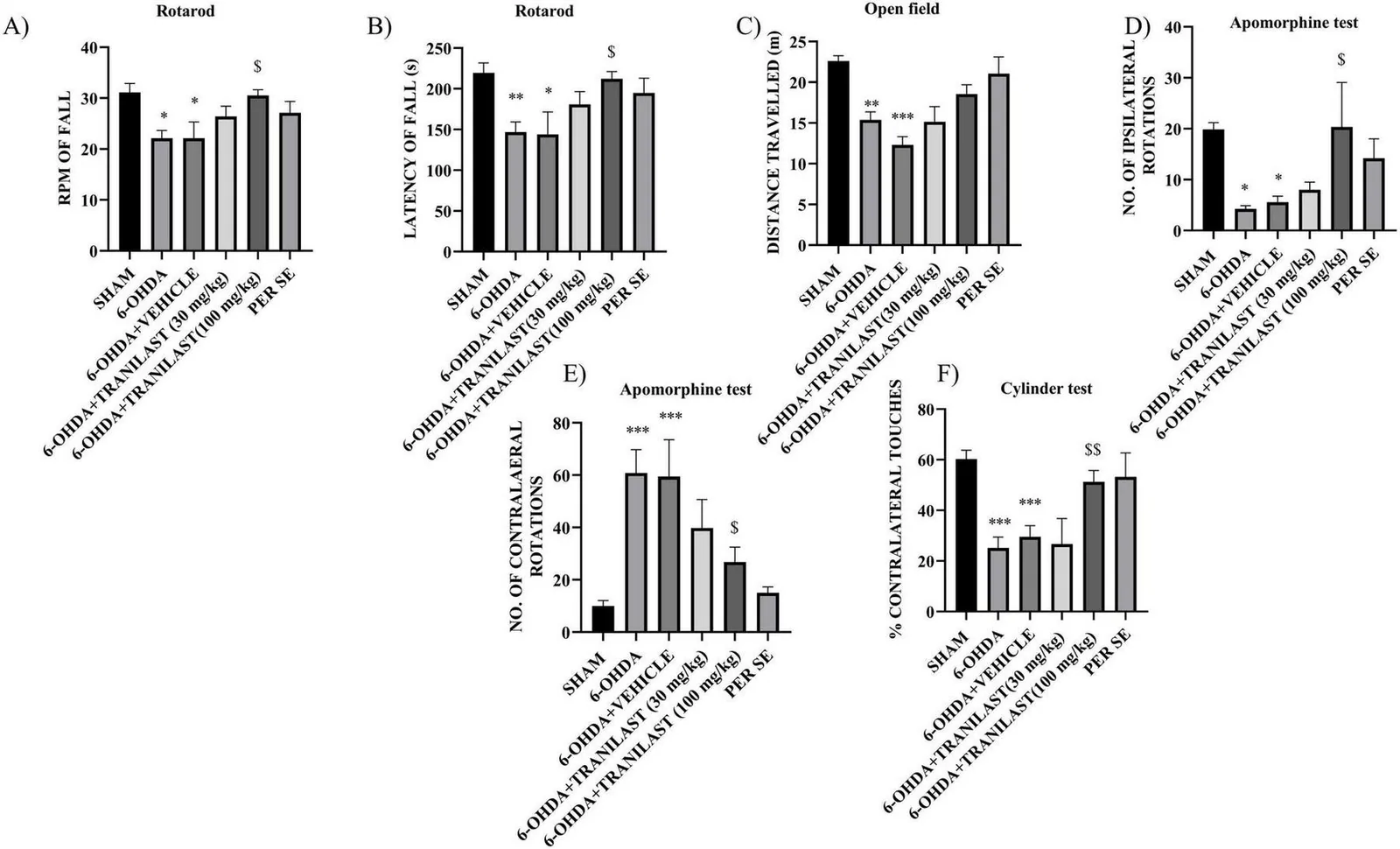
Effect of Tranilast (30 and 100 mg/kg) on locomotor activity in 6-OHDA model of PD. **(A)** Latency of fall (s) and **(B)** RPM of fall in Rotarod test, **(C)** distance travelled by animal (m) in Open field, **(D)** No. of ipsilateral rotations, **(E)** No. of contralateral rotations in Apomorphine induced rotation test and **(F)** % contralateral touches in cylinder test. Values are expressed as Mean ± SEM. *p < 0.05 vs. sham; **p < 0.01 vs. sham; ***p < 0.001 vs. sham; $*p* < 0.05 vs. 6-OHDA; $$*p* < 0.01 vs. 6-OHDA (*n* = 7–8). Sham animals (surgery controls), 6-OHDA-administered animals, 6-OHDA-administered animals treated with either Tranilast (30 or 100 mg/kg) or vehicle and the naïve control animals treated with Tranilast (100 mg/kg) (*Per se*).

The motor function of the animals was evaluated using a rotarod test. In the rotarod test, fall latency and fall rpm were recorded. Disease (*p* < 0.001) and vehicle-treated (*p* < 0.05) animals showed a significant reduction in both these parameters compared to the sham animals. Animals treated with Tranilast (100 mg/kg) (*p* < 0.01) exhibited improved rotarod performance as compared to diseased and vehicle-treated animals, while a 30 mg/kg dose of Tranilast did not show any significant results (Figures 2A, B). Similarly, a significant reduction was observed in the open field performance of the 6-OHDA rats (*p* < 0.01) and the vehicle-treated group (*p* < 0.001). However, the Tranilast treatment resulted in only a modest, non-significant improvement and did not fully reverse the phenotype in this assay (Figure 2C).

A gold standard test of apomorphine-induced rotations for 6-OHDA model evaluation was performed, in which diseased and vehicle-treated animals showed more contralateral rotations as compared to ipsilateral rotations with respect to the sham animals. The effect of Tranilast (30 and 100 mg/kg) on the apomorphine-induced rotation test was assessed. Animals that received a dose of 100 mg/kg showed improvement, exhibiting less contralateral rotation than disease-, vehicle-, and low-dose-treated animals (Figures 2D, E).

Spontaneous forelimb movement was also evaluated using a cylinder test, comparing activity before and after Tranilast treatment (30 and 100 mg/kg) in the diseased animal. Diseased animals exhibited fewer contralateral touches than the sham group of rats (*p* < 0.001). However, a significantly greater number of contralateral touches were made after receiving 100 mg/kg Tranilast (*p* < 0.01) (Figure 2F).

In all these tests, naïve animals with Tranilast (*per se*) did not alter baseline behavioral performance compared with sham animals.

### 3.2 Effect of Tranilast on TH expression

**FIGURE 3:**
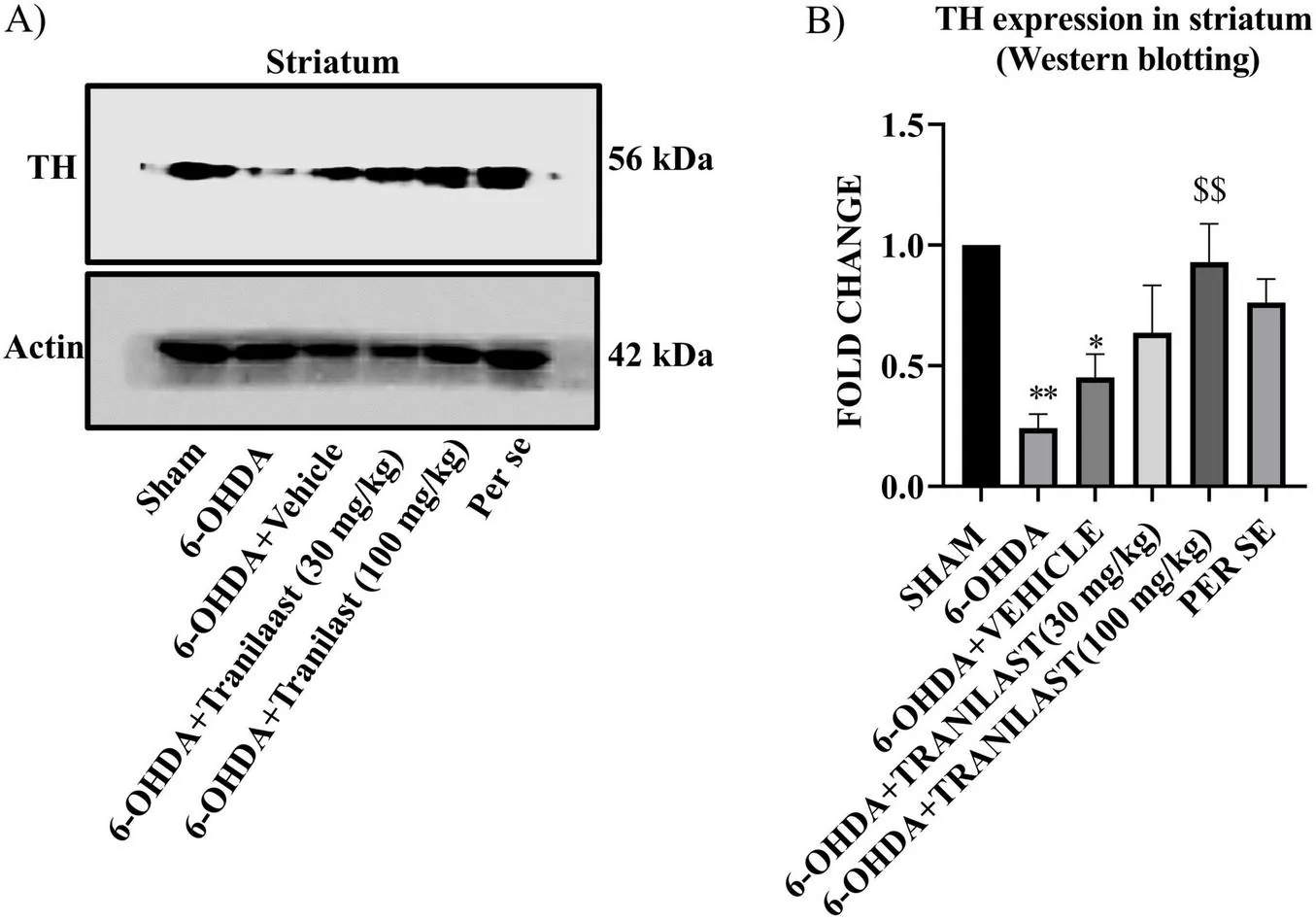
Effect of Tranilast on TH expression in the 6-OHDA model of PD was evaluated by performing western blotting. **(A)** Representative western blot for TH expression in striatum, **(B)** quantification of TH expression in striatum. Values are expressed as Mean ± SEM. *p < 0.05 vs. sham; **p < 0.001 vs. sham; $$***p*** < 0.01 vs. 6-OHDA (***n*** = 5).

Protein expression of TH was quantified using western blotting in the striatal tissue. Significant decreases were observed in the 6-OHDA-treated (*p* < 0.001) and vehicle-treated (*p* < 0.05) groups compared with sham animals. Low-dose Tranilast (30 mg/kg) did not show significant effects, whereas a substantial increase was observed in diseased animals treated with 100 mg/kg Tranilast (*p* < 0.01). Additionally, treatment of naïve animals with Tranilast (*per se*) does not affect TH levels (Figures 3A, B).

The results of immunohistochemistry for TH protein expression were also complemented by western blotting for TH in striatal tissue. A significant decrease was observed in the 6-OHDA-treated and vehicle-treated groups compared to sham animals. Low-dose treatment of Tranilast (30 mg/kg) did not show significant results, but a substantial increase was seen in diseased animals treated with 100 mg/kg of Tranilast. *Per se* animals did not show any difference in TH expression as compared to sham animals (Supplementary Figure 1).

### 3.3 Effect of Tranilast on TRPV2 expression

**FIGURE 4:**
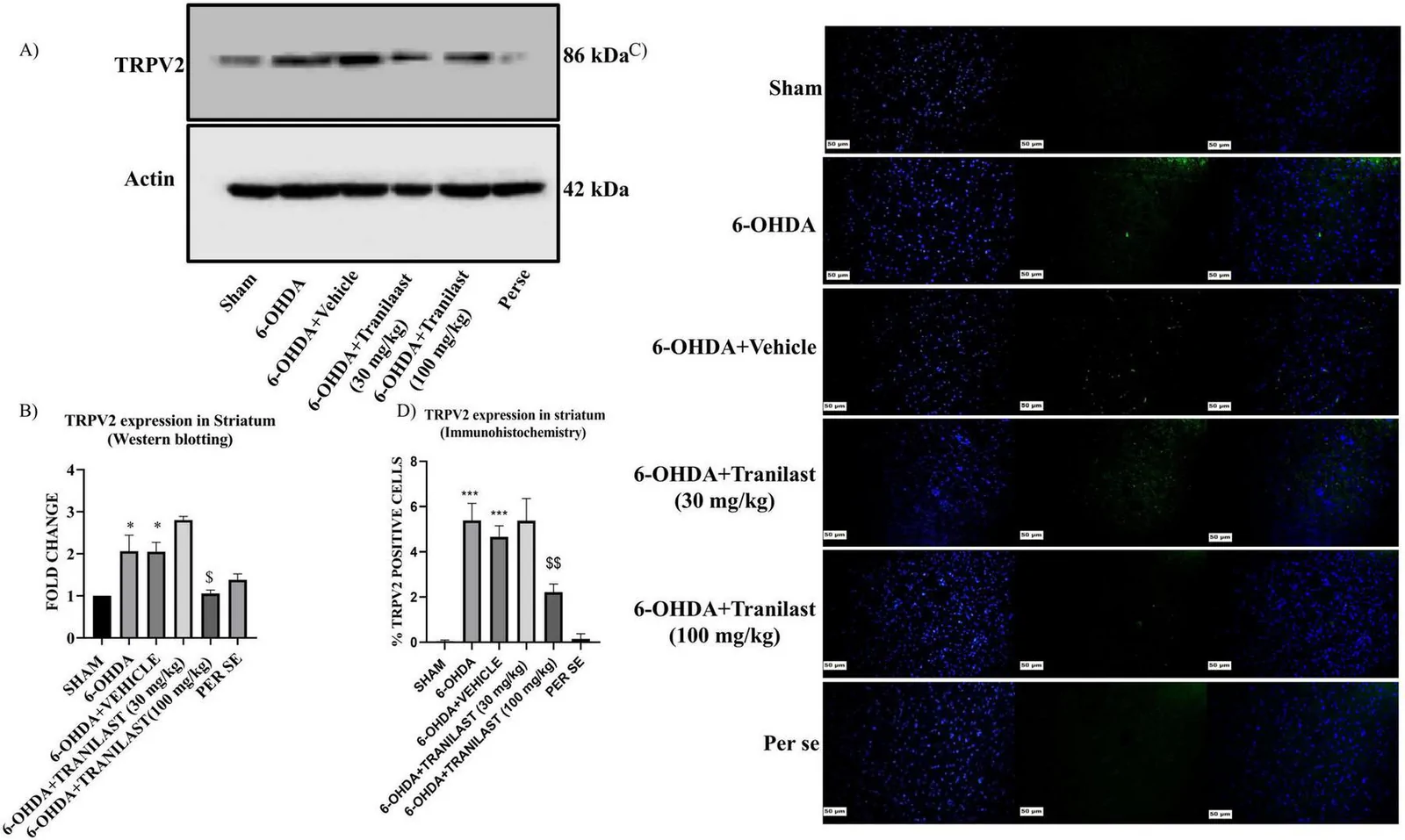
Effect of Tranilast on TRPV2 expression in the 6-OHDA model of PD was evaluated by performing western blotting. **(A)** Immunoblotting for TRPV2 expression in striatum. **(B)** Quantification of TRPV2 expression in striatum. **(C)** Immunofluorescence showing TRPV2 expression in the striatum. **(D)** Quantification of TRPV2 expression in the striatum using immunofluorescence. Values are expressed as Mean ± SEM. *p < 0.05 vs. sham; $***p*** < 0.05 vs. 6-OHDA (***n*** = 3).

Transient receptor potential vanilloid 2 expression levels were compared among groups using western blotting and immunofluorescence assays. Animals in the diseased group (*p* < 0.05, western blotting; *p* < 0.001, immunofluorescence) and the vehicle-treated group (*p* < 0.05, western blotting; *p* < 0.001, immunofluorescence) showed an increased level of TRPV2 in the striatum as compared to the sham group. Treatment with Tranilast at a low dose did not show a significant difference in TRPV2 expression compared with diseased animals, whereas at a higher dose of 100 mg/kg, a significant difference was observed (*p* < 0.05, western blotting; *p* < 0.01, immunofluorescence). Additionally, treatment of naïve animals with Tranilast (*per se*) does not affect baseline TRPV2 levels (Figures 4A–D).

### 3.4 Effect of Tranilast on MDA and GSH levels

**FIGURE 5:**
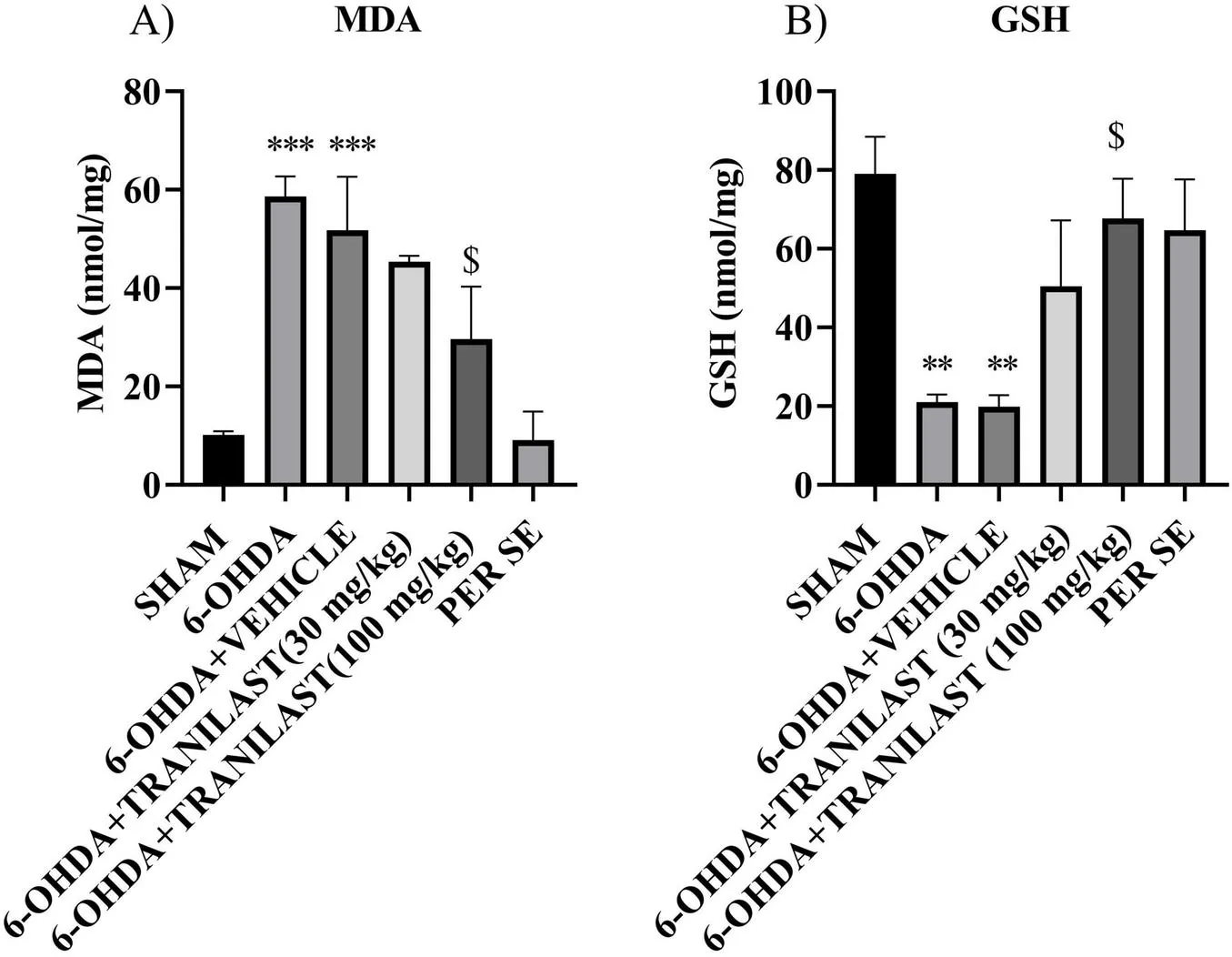
Effect of Tranilast on MDA and GSH level. **(A)** Effect of Tranilast on MDA level, **(B)** effect of Tranilast on GSH level. Values are expressed as Mean ± SEM.**p < <0.01 vs. sham; ***p < <0.001 vs. sham; $*p* < <0.05 vs. 6-OHDA (*n* = 3–5).

Malondialdehyde and GSH levels were estimated and compared across groups, with the diseased and vehicle-treated groups showing increased MDA (*p* < 0.001) and decreased GSH (*p* < 0.01) levels compared with the sham group. Treatment with Tranilast (100 mg/kg) significantly reduced MDA formation (*p* < 0.05) and restored GSH levels (*p* < 0.05), whereas Tranilast at 30 mg/kg had no significant effect. Additionally, treatment of naïve animals with Tranilast (*per se*) does not affect MDA and GSH levels (Figures 5A, B).

### 3.5 Neuroprotective effect of Tranilast on neuronal cell viability

**FIGURE 6:**
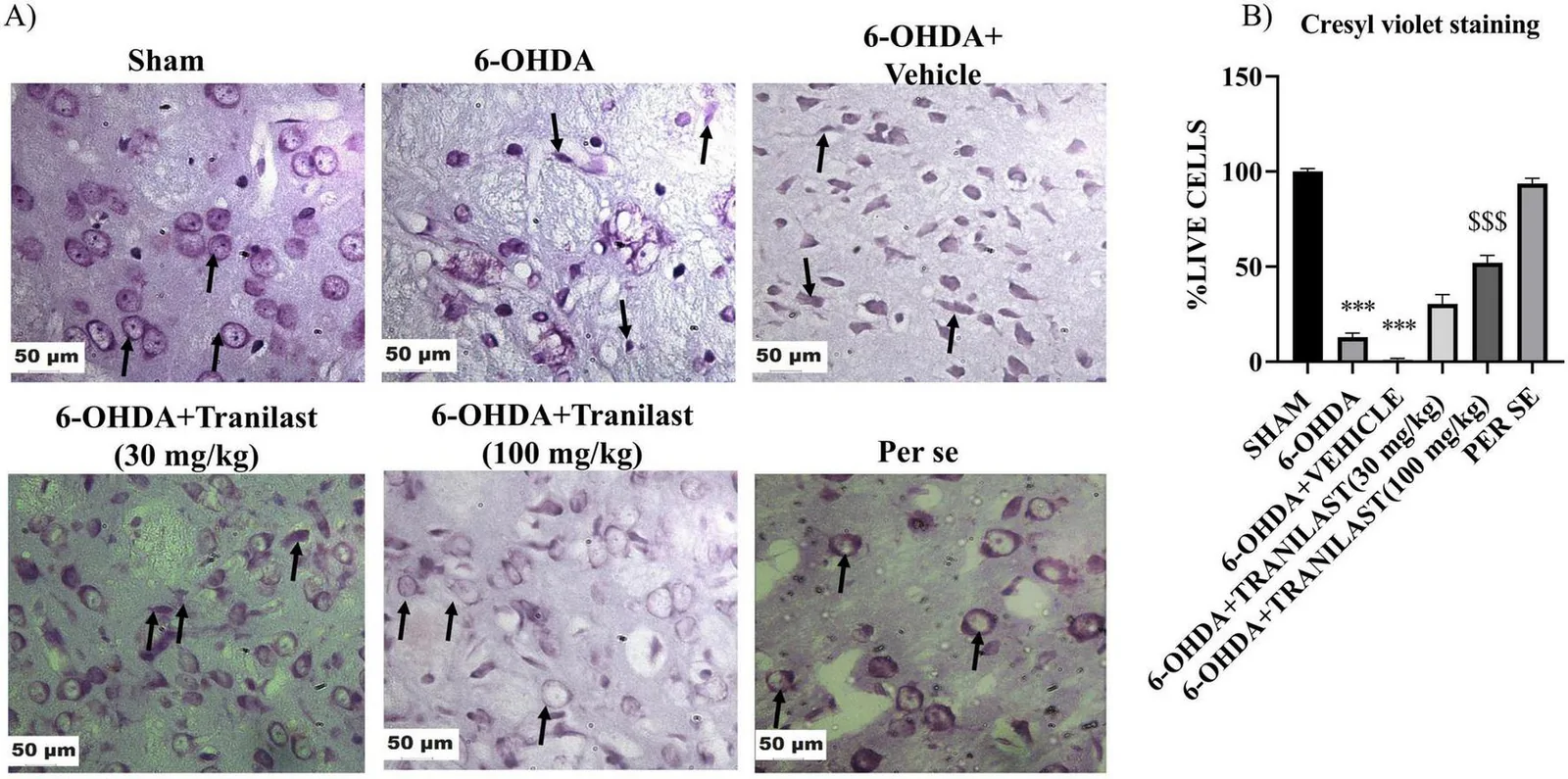
Effect of Tranilast on histopathological parameters. **(A)** Cresyl violet stained brain sections, **(B)** quantification of live cells in each group. Values are expressed as Mean ± SEM.***p < 0.001 vs. sham; $$$*p* < 0.001 vs. 6-OHDA (*n* = 3).

The neuroprotective effect of Tranilast on cell viability was evaluated by cresyl violet staining of striatal brain microsections. Cell morphology was intact in the sham group, whereas a significantly lower (*p* < 0.001) number of live cells was observed in diseased animals and vehicle-treated groups. Treatment with Tranilast (100 mg/kg) significantly reversed the neuronal damage and a significantly increased number of live cells (*p* < 0.001) were seen in this group with respect to the disease group. Finally, no significant difference was observed between the *per se* group and the sham group in the total number of live cells (Figures 6A, B).

### 3.6 Effect of Tranilast on cell viability, cytosolic Ca^2+^ concentration and oxidative stress

**FIGURE 7:**
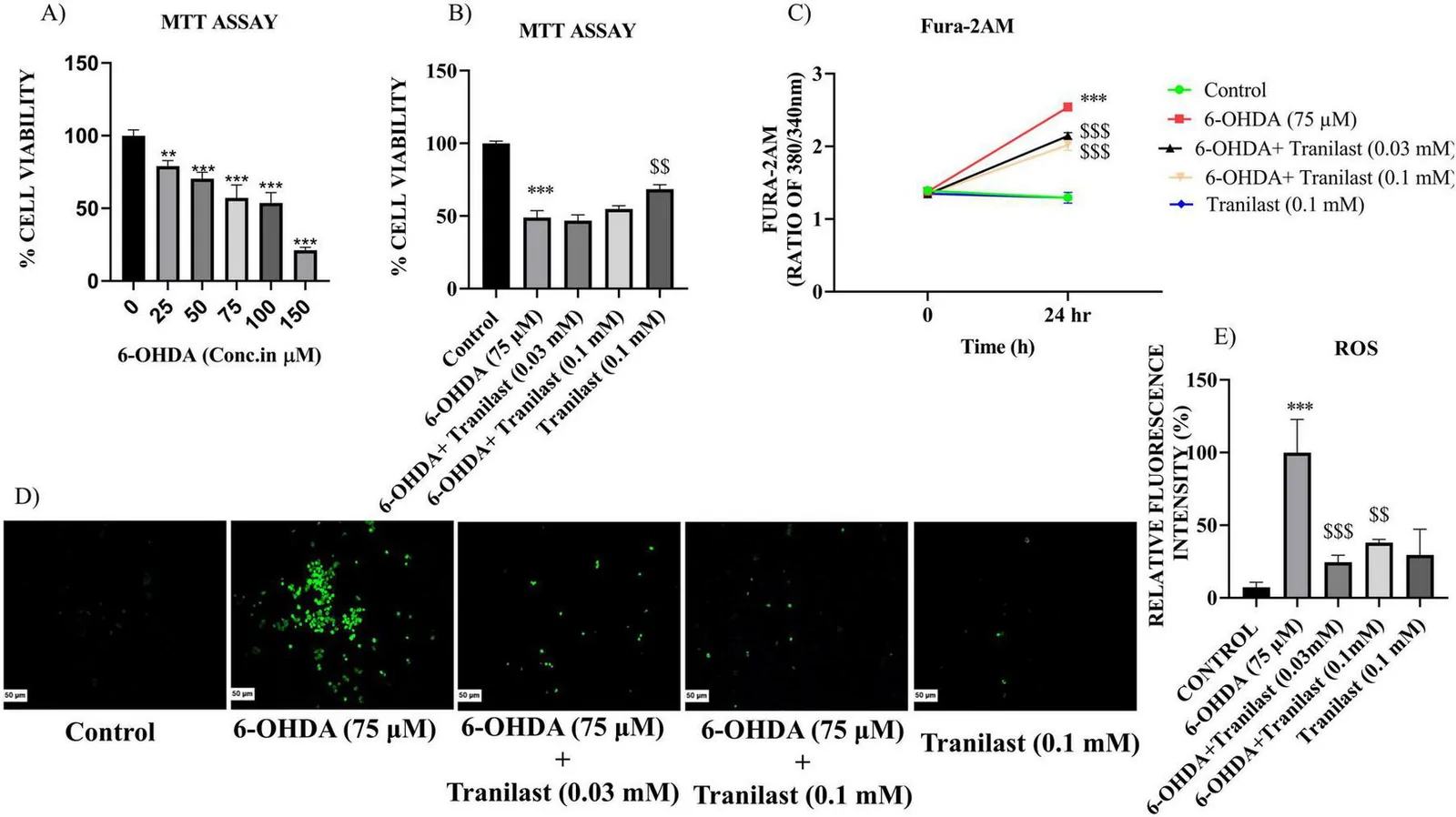
Effect of Tranilast on 6-OHDA treated SH-SY5Y cells. **(A)** Dose-dependent effect of 6-OHDA on SH-SY5Y cells. **(B)** Effect of Tranilast on SH-SY5Y cell viability. **(C)** Effect of Tranilast on Ca^2+^ influx. **(D)** Effect of Tranilast on ROS. **(E)** Quantification of ROS levels for comparison across groups. Values are expressed as Mean ± SEM. **p < 0.01 vs. control; ***p < 0.001 vs. control; $$*p* < 0.01 vs. 6-OHDA; $$$*P* < 0.001 vs. 6-OHDA (*n* = 5).

Firstly, SH-SY5Y cells were treated with different concentrations of 6-OHDA (25–150 μM) to determine the effective working concentration of 6-OHDA (*p* < 0.01, 25 μM; *p* < 0.001, 50–150 μM) (Figure 7A). The SH-SY5Y cells showed a 50 % survival rate at a 75 μM concentration of 6-OHDA, measured by performing an MTT assay and it was used for further experimentation. Co-treatment with Tranilast (0.1 mM) conferred protection against 6-OHDA-induced cell death (*p* < 0.01) (Figure 7B).

Cytosolic Ca^2+^ concentration was measured by using Fura 2-AM dye, and it was found that 6-OHDA-treated SH-SY5Y cells increased levels of Ca^2+^ as compared to the control group (*p* < 0.001). Treatment with 0.03 and 0.1 mM Tranilast significantly suppressed the 6-OHDA-induced elevation in intracellular Ca^2+^ concentration compared with the 6-OHDA-treated group (*p* < 0.001) when fluorescence was measured after 24 h of treatment. Cells treated with Tranilast (0.1 mM) alone showed no change in intracellular Ca^2+^ concentration compared with control cells (Figure 7C).

Finally, ROS generation in SH-SY5Y cells after exposure to 75 μM 6-OHDA was measured using fluorescence microscopy. A significant rise in ROS levels was observed in cells treated with 6-OHDA (*p* < 0.001), which declined when cells were treated with 0.03 and 0.1 mM Tranilast (*p* < 0.001, 0.03 mM; *p* < 0.01, 0.1, 0.1 mM) (Figures 7D, E).

## 4 Discussion

In the present study, we have evaluated the effects of Tranilast in *in-vitro* as well as an *in-vivo* model of PD using 6-OHDA neurotoxin. It has been reported that overproduction of ROS has a negative effect on cellular functions, including apoptotic signaling, mitochondrial functions, and Ca^2+^ homeostasis, which contribute to PD progression (Jahanshahi et al., 2018). These changes in turn produce dopaminergic neuronal death in the brain which causes decreased voluntary movements in PD. In prior investigations done by other researchers using 6-OHDA-induced parkinsonian neurodegeneration, behavioral tests such as the rotational test, open-field test, cylinder test, and rotarod test have been used to measure motor impairments in the animals (Carvalho et al., 2013). Our results evidenced that 2 weeks after the administration of 6-OHDA in the right striatum, adult male rats showed reduced motor activity, which was consistent with earlier reports (Barata-Antunes et al., 2020). These results further suggest that the right lesioned side of the 6-OHDA-treated group showed reductions in tyrosine hydroxylase content, in comparison to the left side (intact hemisphere), which is also in line with earlier studies (Ximenes et al., 2015; Prasad and Hung, 2020).

A rotational test is a well-established, widely used method for assessing the severity of Parkinsonism in the 6-OHDA rat model. Apomorphine-induced rotations are the gold standard test to assess dopamine depletion in the striatum following unilateral striatal lesion of 6-OHDA (Björklund and Dunnett, 2019). In the present study, the apomorphine-induced rotational test revealed significant differences in the numbers of ipsilateral and contralateral rotations following apomorphine administration. These observations result from differential dopamine levels in the striatum that correlate with apomorphine-induced rotational behavior observed in 6-OHDA-treated rats (Barata-Antunes et al., 2020; Prasad and Hung, 2020; Carvalho et al., 2013). However, animals treated with the TRPV2 antagonist Tranilast (100 mg/kg) exhibited fewer contralateral rotations, suggesting fewer dopaminergic receptors available for apomorphine binding. This could be due to reduced damage to dopaminergic neurons, attributed to the effect of Tranilast, which might diminishes Ca^2+^ influx and thus maintains cellular homeostasis.

The Rotarod test assesses motor performance and motor learning in rodents (Shiotsuki et al., 2010). In 6-OHDA toxin-treated groups, fall time (latency of fall) and fall rpm were significantly lower than in the sham group. The administration of 6-OHDA decreased striatal dopamine content, which is closely related to tyrosine hydroxylase-positive neuronal death in the striatum, and reduced latency on the rotarod test. TH is a prominent marker in PD, indicating dopamine synthesis in a particular brain region (Daubner et al., 2011). This is further confirmed by the cylinder test, in which the lesioned group exhibited contralateral motor deficits, as evidenced by a progressive reduction in contralateral forelimb touches on the cylinder wall over time, compared with the respective control groups (Barata-Antunes et al., 2020). Tranilast reversed motor impairment and TH expression at a dose of 100 mg/kg in 6-OHDA-induced parkinsonian neurodegeneration, as confirmed by immunoblotting and immunohistochemistry (Hernandez-Baltazar et al., 2017).

In total, although we observed improvements in coordinated motor performance and forelimb asymmetry, there was a minimal, non-significant improvement in total locomotor activity in the open-field test. This may be because the open-field activity is a more general measure of spontaneous exploratory locomotion that may also be affected by motivation, anxiety-related behavior, habituation and compensatory neural mechanisms, whereas the rotarod and cylinder tests are particularly sensitive to motor co-ordination and unilateral nigrostriatal dysfunction, respectively. In addition, a study by Chen et al. (2019) has attributed these changes to differences in dopamine content. It was demonstrated that forced behaviors, such as rotarod performance, can be partially maintained even when DA release is severely compromised, whereas spontaneous behaviors, such as open-field activity, are much more sensitive to impaired DA release. Therefore, a partial reversal of dopaminergic deficits, as reflected by TH levels, might rescue rotarod deficits but might still be insufficient to reverse open-field activity, which would probably require a greater degree of dopaminergic restoration.

Thereafter, the effect of Tranilast on TRPV2 expression was also evaluated. Based on previous studies, TRPV2 is among the many TRPs found to be responsible for maintaining Ca^2+^ homeostasis, which is one of the key factors causing CNS diseases (Enrich-Bengoa et al., 2022). In the present study, TRPV2 expression was higher in 6-OHDA-treated animals than in sham and Tranilast (100 mg/kg) treated groups. However, because TRPV2 activity is also regulated by stimulus-dependent translocation from intracellular compartments to the plasma membrane, the present findings cannot determine whether 6-OHDA primarily alters total TRPV2 abundance or merely its translocation. Future studies using approaches such as cell-surface biotinylation or high-resolution co-localization imaging with plasma membrane markers would help characterize the role of TRPV2 in PD mechanistically (Cui et al., 2022).

Despite these limitations, because TRPV2 expression was reduced following Tranilast administration, we expected reduced calcium influx into the cell. It was tested using the Fura-2 AM assay. It was shown that in SH-SY5Y cells, 6-OHDA increased Ca^2+^ influx at the 24h time point. However, inhibition of TRPV2 channels by Tranilast reduced calcium entry in these cells. Similar reductions have been observed *in vitro* in prior studies involving trainlast in CNS disease models (Jahanshahi et al., 2018; Thapak et al., 2020). Thereafter, reduced calcium levels could further prevent damage to cellular organelles. In the present study, these changes were accompanied by elevation in MDA and reduction in GSH levels in 6-OHDA animals compared with sham animals. Treatment with Tranilast showed promising results in decreasing MDA levels and increasing GSH levels, and hence restoring antioxidant balance (Miyachi et al., 1987; Cui et al., 2022; Darakhshan and Pour, 2015). However, it is also possible that Tranilast could act in part on other channels and intracellular stores, which should be considered in future investigations. Moreover, though sample sizes were calculated based on the power analysis of our prior studies involving TRP channels, we have relatively small sample sizes for some of the histopathological and molecular estimations.

Overall results of this study suggest that TRPV2 is a potential therapeutic target for PD. Besides, it demonstrates the neuroprotective potential of Tranilast in PD, but more detailed research is needed before considering Tranilast as a possible treatment option for PD. It is also worth considering that Tranilast exhibits broader pharmacological activities beyond TRPV2 inhibition, including effects on NLRP3 inflammasome signaling, TGF-β signaling and NF-κB activation pathways (Saeedi-Boroujeni et al., 2021; Zhuo and Zhuo, 2019; Takahashi et al., 2020). In the future, the use of alternative genetic approaches, such as TRPV2 knockdown, overexpression, knockout, or rescue experiments, could more directly establish its mechanistic contribution to the observed neuroprotective effects. Additionally, a more comprehensive neuropathological characterization using additional markers of neurodegeneration, gliosis, neuroinflammation, or apoptosis would be beneficial in the future. Nevertheless, our study establishes a paradigm for future investigations into the beneficial effects of Tranilast in PD and other neurodegenerative disorders. Additionally, the findings of the present study should only be interpreted in the context of the 6-OHDA model of PD. Although it is a well-established PD model, it doesn’t recapitulate some key pathological features of human PD, such as alpha-synuclein aggregation, Lewy body formation, progressive disease evolution, and non-motor manifestations (Prates-Rodrigues et al., 2025). Therefore, validation of TRPV2-associated changes should also be done in other complementary model systems, particularly α-synuclein-based models, to further establish the relevance of this pathway across different aspects of PD pathology. Furthermore, *in vitro* experiments were done in undifferentiated SH-SY5Y cells in line with prior investigations (Vaidya et al., 2022b,^2025^). The experimental results could be further validated in other physiologically relevant neuronal model systems, such as primary midbrain dopaminergic cultures or iPSC-derived dopaminergic neurons, to strengthen their overall relevance.

Finally, as Tranilast is already approved for use in countries like Japan and South Korea, it seems to be safe for human consumption and could hence be repurposed for neurodegenerative disorders in the coming years (Pae et al., 2008).

## Data Availability

The raw data supporting the conclusions of this article will be made available by the authors, without undue reservation.

## Supplementary material

Supplementary Figure 1 Effect of Tranilast on TH expression determined using IHC. **(A)** Representative IHC images showing TH staining in the right striatum across different groups.
